# Identification and Manipulation of the Molecular Determinants Influencing Poliovirus Recombination

**DOI:** 10.1371/journal.ppat.1003164

**Published:** 2013-02-07

**Authors:** Charles Runckel, Oscar Westesson, Raul Andino, Joseph L. DeRisi

**Affiliations:** 1 Howard Hughes Medical Institute, Bethesda, Maryland, United States of America; 2 Department of Biochemistry and Biophysics, University of California San Francisco, San Francisco, California, United States of America; 3 University of California Berkeley/San Francisco Joint Graduate Group in Bioengineering, San Francisco, California, United States of America; 4 Department of Microbiology and Immunology, University of California San Francisco, San Francisco, California, United States of America; Washington University School of Medicine, United States of America

## Abstract

The control and prevention of communicable disease is directly impacted by the genetic mutability of the underlying etiological agents. In the case of RNA viruses, genetic recombination may impact public health by facilitating the generation of new viral strains with altered phenotypes and by compromising the genetic stability of live attenuated vaccines. The landscape of homologous recombination within a given RNA viral genome is thought to be influenced by several factors; however, a complete understanding of the genetic determinants of recombination is lacking. Here, we utilize gene synthesis and deep sequencing to create a detailed recombination map of the poliovirus 1 coding region. We identified over 50 thousand breakpoints throughout the genome, and we show the majority of breakpoints to be concentrated in a small number of specific “hotspots,” including those associated with known or predicted RNA secondary structures. Nucleotide base composition was also found to be associated with recombination frequency, suggesting that recombination is modulated across the genome by predictable and alterable motifs. We tested the predictive utility of the nucleotide base composition association by generating an artificial hotspot in the poliovirus genome. Our results imply that modification of these motifs could be extended to whole genome re-designs for the development of recombination-deficient, genetically stable live vaccine strains.

## Author Summary

Viral recombination is critical to understanding the evolution of viral groups and impacts vaccine design, but is poorly understood. In the poliovirus vaccine, recombination is one potential mode of failure where vaccine strains recombine to produce a pathogenic product. We combine gene synthesis and deep sequencing to generate a high-resolution recombination map of poliovirus, both as a model RNA virus and a continuing threat that has yet to be eradicated. This map shows that recombination is concentrated into hotspots and suggests that predictable and alterable motifs in the RNA sequence are associated with recombination frequency. We demonstrate the utility of these observations by re-designing a poliovirus strain to recombine more frequently than normal, facilitating future studies on the role of viral recombination during infection. This result suggests that a large-scale redesign of the entire poliovirus genome to dampen recombination may be feasible, with implications for producing safer and more stable live vaccines.

## Introduction

Recombination in RNA viruses is a source of genetic diversity and rapid evolutionary change and may result in the emergence of new strains by facilitating shifts in cell tropism, antigen profile and pathogenicity. The mechanism of RNA virus recombination can proceed through re-assortment of genome segments, as is the case for the Influenza A virus, or through the generation of chimeric viral genomes during replication for non-segmented viruses. This recombination is frequent in the wild with different recombinant genotypes rising to dominance and declining over a timescale of only a few years [1]. Sequencing of large numbers of viral isolates has revealed instances of intra-species recombination in many human-infecting RNA viruses with major public health implications, including norovirus [2], astrovirus [3], flavivirus [4] and at least eight species of picornavirus [5]–[10]. Rare inter-species recombinants, such as the enteroviruses HEV90 [11] and HEV109 [12], have also been described.

Viral recombination not only impacts public health by the evolution of new viral strains, but may also undermine live-attenuated vaccines by producing a pathogenic strain derived from the attenuated strains. The oral poliovirus vaccine (OPV) is the most famous example, where three attenuated serotypes of poliovirus are typically administered simultaneously. One week after inoculation, over a third of Sabin-2 and Sabin-3 viruses shed are recombinant [13]. In the worst case, recipients can develop vaccine-associated paralytic poliomyelitis, potentially through a recombined strain. Vaccine derived polioviruses (VDPVs) may also recombine with other circulating strains of enterovirus to create pathogenic chimeras [14]. Such events have caused outbreaks in numerous locations [15]–[18] and remain an ever-present consideration for newly designed live attenuated vaccines, such as the recently proposed tetravalent Dengue virus vaccine [19]. For engineered vaccine strains, a greater understanding of the underlying molecular determinants influencing recombination in RNA viruses has the potential to mitigate unwanted outcomes.

Besides its global health importance, poliovirus has also long served as a model RNA virus and in particular as a model system for the study of recombination. Viral recombination was first demonstrated in poliovirus [20], subsequently confirmed biochemically [21] and there have been extensive studies since in cell culture examining the timing and topology of recombination between different serotypes and between nearly identical construct strains [22]–[26]. A genetic map of poliovirus using temperature-sensitive mutants first determined the location of the capsid and polymerase genes [27]. Recombination among poliovirus strains in the wild have been readily observed and provide further opportunity for post hoc genetic analysis [17]. Long-term infection of an immune-compromised individual demonstrated that viruses derived from a single lineage also recombine during an infection, but are usually undetectable due to a lack of markers [28]. Together, cell culture and phylogenetic studies have indicated that recombination is not randomly distributed through the genome [26], [29]. A model for the mechanism of poliovirus recombination was proposed by Kirkegaard and Baltimore (1986). Briefly, the “template-switch” model consists of premature termination of replication and association of the nascent strand with a different template genome, followed by a resumption of replication yielding a chimeric daughter genome. Consistent with this template-switch model, nucleotide homology between viral species may be a major determinant of recombination frequency [22].

Protein incompatibility has also been suggested to constrain the generation of viable recombinants. For example, recombination between the genes encoding the interlocking capsid proteins has rarely been observed [5], [8], [30]. However, a lower frequency of recombination in the genes encoding structural proteins may also be the result of differing levels of nucleotide similarity, since capsid genes tend to possess greater sequence diversity than the non-structural genes [8].

The effects of RNA secondary structure add yet another confounding element to the analysis. Enterovirus genomes possess well-documented RNA secondary structures that have been associated with recombination breakpoints [31], [32], however it is difficult to disentangle the relative contributions of nucleotide identity and the secondary structure itself with respect to recombination, especially since the sequences of these structures are highly conserved [33], [34].

In efforts to overcome these issues, previous cell-culture studies have employed nearly identical strains with selectable markers, restriction-enzyme specific mutations [25], [26], or unique PCR-primer annealing sites [23], [24] to detect recombination events over parts of the poliovirus genome at an effective resolution of ∼500–1000 nt. It has been estimated from these studies that the frequency of recombinant progeny arising from a single passage of two co-cultured strains is roughly 1-20% [22]–[26] and some studies have indicated that the relative recombination frequency varies in different regions of the genome, with the structural genes having a lower frequency than the non-structural genes [25], [26].

In order to obtain a higher resolution map and to elucidate the sequence-specific determinants underlying poliovirus recombination, we have developed an approach utilizing a synthetic poliovirus genome engineered to contain 368 specific markers. By ultra deep sequencing, we examined the resulting viral population produced by co-infection of cells with wild type and synthetic poliovirus genomes. The resulting high-resolution map of recombination frequencies allowed us to uncover key genomic features that both enhance or repress recombination. Based on these results, we then reengineered a portion of the genome to increase the frequency of recombination. These results identify RNA features influencing recombination and demonstrate that they may be altered with predictable outcomes. These results also suggest possible routes to attenuating recombination frequencies in synthetic vaccine strains.

## Results

### Construct Strain Design and Validation

**Figure 1:**
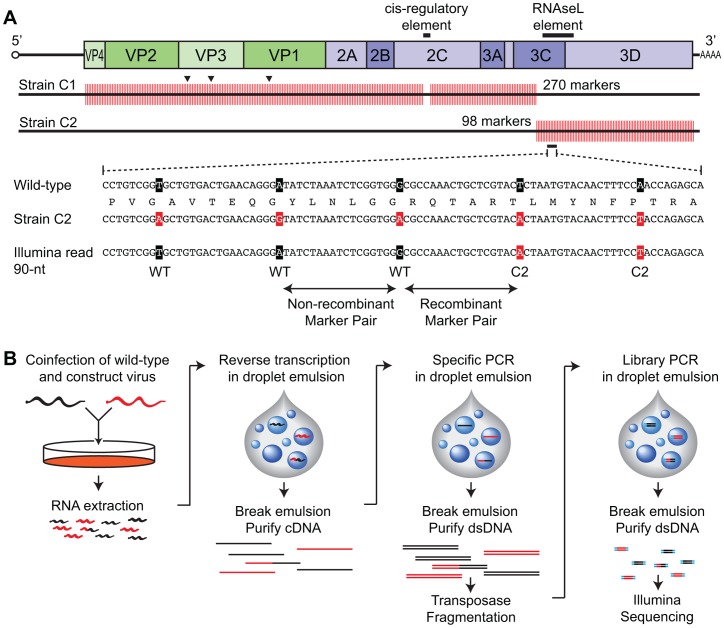
Experimental overview. A. Synonymous mutations were made in a synthetic poliovirus 1 genome every 18 nt. Construct and wild-type plasmid DNA was exchanged to create two partially tagged strains, C1 and C2. Mutations observed in recovered populations are indicated by arrows in C1. Recombination (or a lack of recombination) is determined by Illumina sequencing, with recombination rate calculated as the ratio of discordant to concordant marker pairs at any given location. B. Wild-type and construct viruses co-infect a HeLa monolayer at high MOI. RNA is extracted after the infectious cycle is complete and reverse transcribed in an oil droplet emulsion. The emulsion is broken and the cDNA is amplified to ∼2.6 kb PCR amplicons in another emulsion. This emulsion is broken and recovered large PCR amplicons are fragmented and adapters ligated to the sheared ends by transposase. Illumina compatible fragments are again amplified by PCR in emulsion prior to extraction, quantitation and Illumina sequencing.

Gene synthesis is inherently free from the limitations of traditional site directed mutagenesis and cloning procedures and thus enables any number of genetic modifications. Using gene synthesis, we have designed and synthesized a poliovirus genome engineered explicitly for the purpose of measuring enterovirus recombination. In total, we specified 368 synonymous marker mutations, spaced every 18nt, spanning the poliovirus 1 coding region (Figure 1A) with the intent of using Illumina deep sequencing technology to detect recombinants between wild type and mutant poliovirus. This synthetic genome was chemically synthesized (Blue Heron, Inc.) and then tested for viability by transfection.

The initial full-length synthetic mutant virus construct was not viable when transfected into HeLaS3 cells. Therefore, we chose to arbitrarily divide the parental synthetic construct into two sub-constructs (C1 and C2, Figure 1A and M&M). These constructs were viable and were passaged twice after transfection to allow any adaptation that was necessary and to generate large quantities of virus for further analysis of a single stock. Passage 2 viruses (P2) achieved CPE in a similar time as wild type and with similar burst sizes and titers (Table S1). To check for additional mutations or reversion of our engineered markers, the genomes of both constructs were recovered and re-sequenced after three passages. No reversions were detected, however three additional mutations were revealed, all in the capsid region (G1872U, U2134C and A2663G) of C1 (black triangles, Figure 1A). No mutations were observed in C2. One-step growth curves of the constructs reveal robust amplification for the C2 strain compared to wild-type, while the C1 strain was consistently slower than wild-type by 5–10-fold at 4, 6 and 8 hours post infection. However, C1 ultimately produced a similar number of competent infectious virions (∼10^9^ pfu/mL) as wild type by 10 hours and beyond (Figure S1A). Plaque size and morphology were similar between strains (Figure S1B) and direct competition assays, where viruses were co-inoculated at equal titer and allowed to compete, showed nearly equivalent representation after one passage. In contrast, by passage 4, the wild type had completely out-competed both synthetic construct strains (Figure S1C). These results indicate that there was a mild loss of fitness incurred by the synonymous mutations in the synthetic construct strains, yet they were viable and competitive with the wild-type strain for at least one infectious cycle. These results are consistent with previous observations [35], [36].

### Recombination Mapping by Deep Sequencing

Monolayers of HeLaS3 cells were coinfected with wild-type virus and each of the synthetic construct strains at a multiplicity of infection (MOI) of 10 PFUs/cell. Viral RNA was harvested after 24 hours. Relative levels of viral RNA was determined by strain-independent PCR/cloning/strain-specific colony PCR. We observed a WT:Construct RNA ratio of 0.61–0.92 before inoculation and 0.75–1.08 at harvest. Illumina-compatible libraries were generated from the RNA using a standard protocol intended for RNA-seq meta-transcriptome applications [37]. A “no coinfection” control was conducted in parallel, wherein cells were infected with each virus in separate cultures, harvested at 24 hr and pooled prior to library generation. The no-coinfection control libraries provide a measure of the false-positive rate, since these samples were cultured separately and thus do not contain any recombinant virus. However, we observed high rates (1.5 breakpoints per genome) of recombinant sequences in the dataset, presumably caused by template switching during reverse transcription and/or PCR [38], [39]. To circumvent the occurrence of false-recombination during library preparation, we employed a serial oil/water-emulsion droplet technique to effectively create single molecule reaction vessels for all subsequent enzymatic operations [40]. Each step of the process, beginning with reverse transcription and proceeding through fragment amplification and Illumina adaptor PCR, was conducted within separate emulsions as diagrammed in Figure 1C. After optimization of the library preparation, biological replicates of the coinfection experiment and matching no-coinfection controls were prepared and sequenced using an Illumina HiSeq2000.

### Data Analysis

The error rate of Illumina sequencing and the error rate of enzymatic amplification both present challenges for the interpretation of recombination mapping data. With previously reported recombination frequencies of 1–20% per genome per infectious cycle and 366 marker pairs, the mean ratio of recombinant to non-recombinant marker pairs is expected to be 1 in 10^4^ to 1 in 10^3^. Using published enzyme error rates, the highest fidelity commercially available enzymes possess a theoretical error rate of 1∶40,000 [40], [41]. Illumina sequencing has published error rates per base of 0.1–1% [42]. To surmount both of these confounding sources of error, we only designated a read as evidence of a recombination breakpoint if, and only if, the candidate breakpoint was supported by a minimum of two markers on each side (Figure 1B). This requirement effectively squares the overall error rate at a cost of approximately 50% of the data set.

After quality filtering by removing reads of with any ambiguous base calls (Ns) and trimming 10 nt off of the error-prone 3′ end of each read, 75 and 66 million reads (each now 90 nt long) were obtained for the biological replicates, yielding a total of 110.8 and 99.0 million marker pairs mapped, disallowing any mismatches or ambiguities in alignment (Table 1). Marker pairs within 40 nt of amplicon primer binding sites were also removed, in addition to those modified for RFLP analysis (see M&M). In total, 82% of marker pairs passed all quality thresholds and were used for this analysis. Relative RNA abundance confirmed a WT:Construct RNA ratio of 0.78 and 0.82. The signal-to-noise ratio of the coinfection to the no-coinfection control, defined as the sum of recombination frequencies observed at each marker pair in the experimental dataset divided by the no-coinfection control, ranged from 23.1∶1 to 29.5∶1, and averaged 26.6∶1. While the biological replicates were highly correlated (R^2^ = 0.72, Figure S2A), there was no similarity to the no-coinfection control (R^2^ = 0.10) as expected. The replicates of the no-coinfection controls exhibited weak similarity (R^2^ = 0.24). We observed a 2-fold variation in per marker pair recombination frequency between replicates, however the rank order of marker pairs was highly similar (Spearman ρ = 0.91, Figure S2B) thus permitting identification of associations despite small differences in magnitude.

**Table 1:** Mapping statistics.

	Replicate 1	Replicate 2
Reads mapped	74,891,647	65,738,764
Marker pairs mapped	110,815,512	98,986,069
Genome equivalents mapped (mapped pairs/290 pairs)	382,122	341,331
Recombinant sequences observed	31,410	26,336
Wild-type : Construct Reads	0.78	0.82
Recombination rate (sum of observed per marker pair fraction recombinant)	0.117	0.101
Control recombination rate	0.00396	0.00437
Signal-to-noise ratio	29.5∶1	23.1∶1

### Overall Topology of Recombination

**Figure 2:**
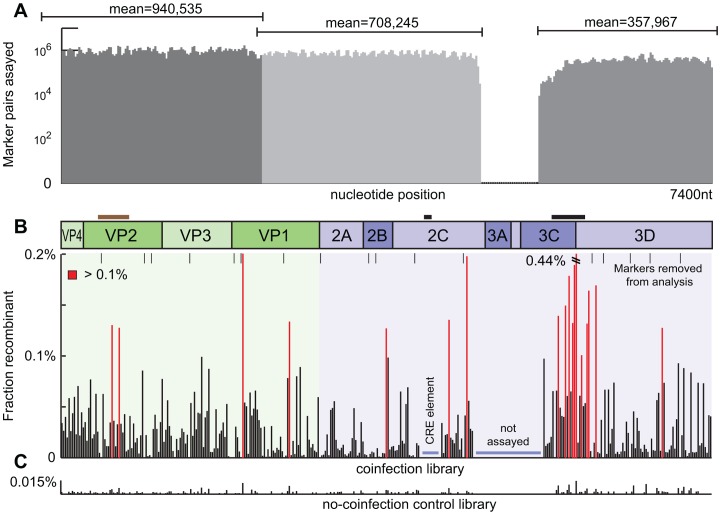
Recombination map. A. Sequencing coverage depth per marker pair. B. Frequency of discordant vs. concordant marker pairs across the genome. Positions of high recombination (over 0.001 discordant) in red. Not assayed areas marked in blue (see Methods). C. Individually infected virus strains were pooled after RNA extraction to determine false recombination from the library preparation steps, displayed at the same scale as section B.

We observed over 50,000 recombinant molecules in this mapping experiment (Figure 2). The overall distribution of recombination breakpoints was highly consolidated with 47% of the total breakpoints observed in only 10% of the marker pairs with a mean recombination frequency of 0.14% versus 0.024% in the lower 90% of marker pairs. Breakpoint occurrences were observed between all but two of the marker pairs, with no significant difference between capsid and non-structural genes when considering mean or median recombination frequencies averaged over those regions (0.031% vs. 0.042% mean crossovers per 17-nt, p>0.1). Gene boundaries have been proposed as recombination hotspots [43], however no association was observed examining either the precise site of gene boundaries, or those sites and their adjacent marker pairs. The total recombination frequencies measured were 10% and 12% for the biological replicates respectively. These frequencies are within and favoring the upper bound of previous estimates [22]–[24], [44].

### RNA Secondary Structure

**Figure 3:**
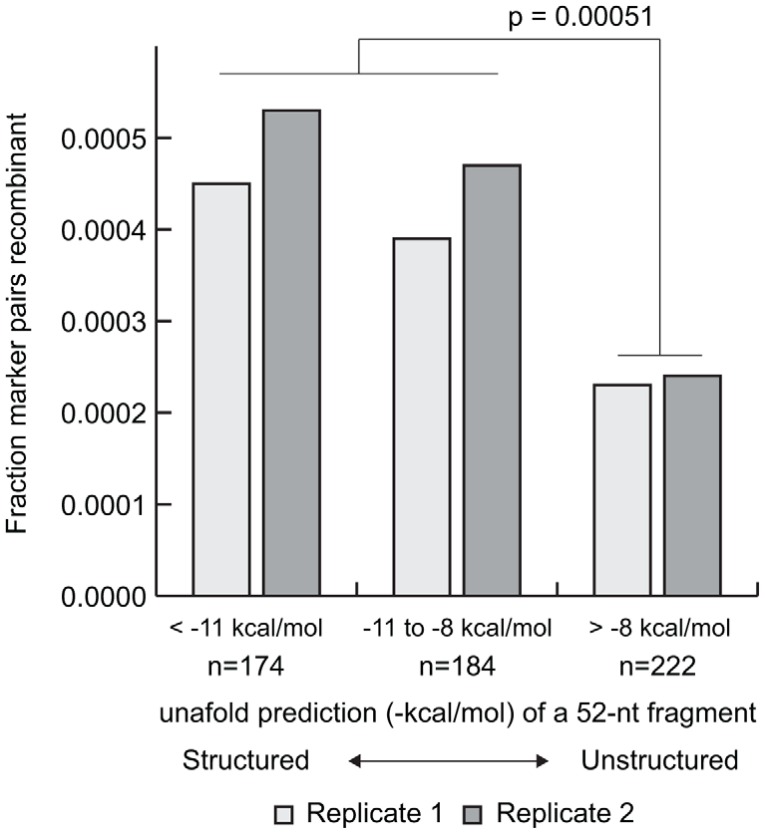
Predicted secondary structure is associated with recombination frequency. Marker pairs are binned based on the Unafold calculated RNA folding energy of the marker pair and the flanking pairs (52-nt fragments). Biological replicates are shown in black and grey. Statistical associations are determined by Student's t-test after multiple testing correction.

RNA secondary structure has previously been identified as an enhancer of recombination and our results strongly support this association [29]. The largest peak of recombination frequency coincides with the RNAseL element (p<10^−6^), an RNA secondary structure located in the 3C gene and associated with host nuclease inhibition [34]. Recombination over this element was 3.5 times higher than the rest of the genome and included the largest recombination hotspot observed (0.44% recombinant)(Figure 2A). The CRE element, the only other well characterized RNA structure in the coding region [45], was not modified in our synthetic constructs due to concerns over viability of the mutant. We examined predicted secondary structure over the entire genome using Unafold [46] and a sliding 52-nt window corresponding to each marker pair and the adjacent marker pairs. Windows with a predicted folding energy of less than −8 kcal/mol associated significantly (p = 0.0005) with reduced recombination frequency (Figure 3), with structured regions exhibiting a higher rate of recombination.

### Sequence Composition

**Figure 4:**
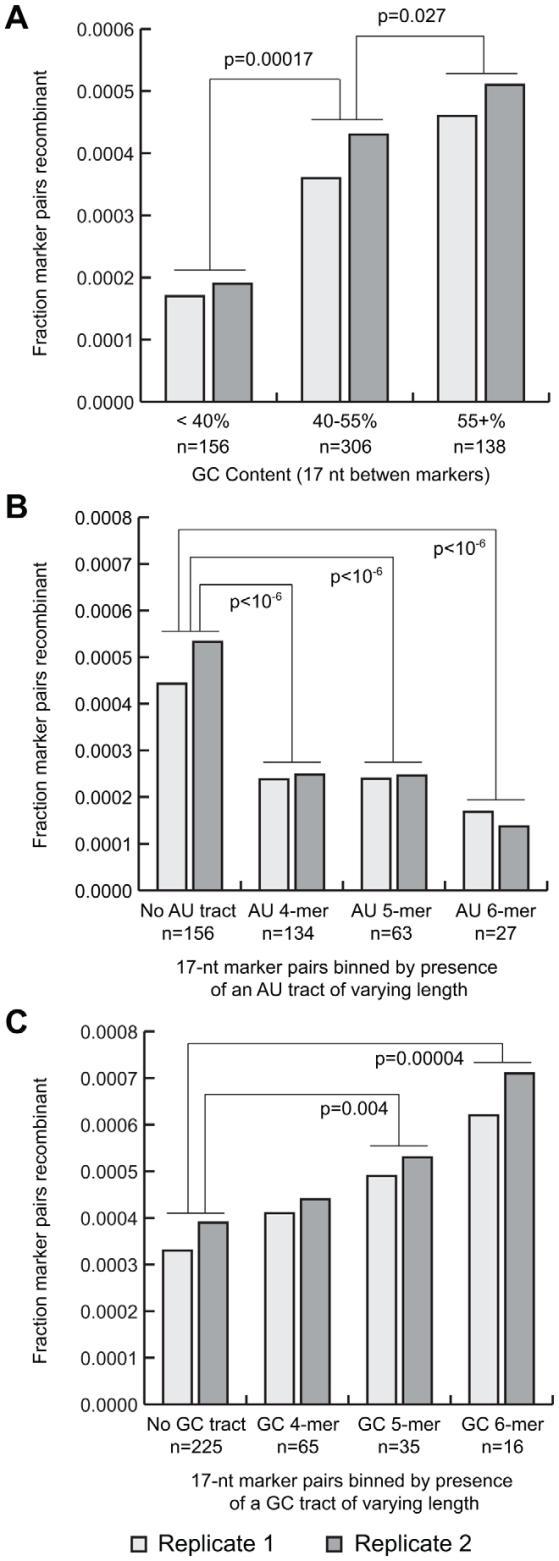
GC content is associated with higher recombination frequency. A. Marker pairs are binned by the GC content of the intervening 17 nt. B. Marker pairs are binned by the presence of tracts of consecutive A or U nucleotides of varying lengths. Bins are non-exclusive (i.e. marker pairs in the AU 6-mer bin are also included in the 4-mer bin). C. As B, binned by the presence of G or C tracts.

We examined associations between sequence composition and recombination frequency. We found that GC content bias was associated with recombination: high GC marker pairs (>55% over a 17 nt window) were associated with a 1.3x increase in recombination frequency (p = 0.027) and low GC content (<40%) was associated with a 2.1x decrease (p = 0.00017) (Figure 4A). Tracts of AU or GC nucleotides were also associated with reduced and increased recombination, respectively (Figure 4B,C). The magnitude of the effect increased with the length of the tract, from an increase of 1.3-fold for GC tetramers to 1.9-fold for hexamers (p = 0.00004). AU tracts showed an inverse effect, with AU tetramers associated with 2.6-fold reduction in recombination and 3.9-fold for hexamers (p<10^−6^). Effects were observed for GC content even after accounting for AU and GC tracts, and vice versa, suggesting that the two may have independent effects or that the effects are related and involve a more complicated relationship than can be determined with this data set. The “no-coinfection” control data was subjected to the same analysis, however none of the models achieved statistical significance. We also applied these analyses to the dataset shifted one marker pair up or downstream to identify effects that may not manifest themselves locally; no significant associations were observed.

Other motifs were also examined with regard to recombination frequency. Homopolymer tracts and all dinucleotide pairs were compared with no significant associations except for AU and GC tracts; and their associated homopolymers lacked sufficient occurrences to achieve significance. No association was observed between the overall complexity of the sequence between marker pairs, as measured by LZW compression score [47], [48]. To investigate whether more complex or cryptic sequence motifs were associated with recombination frequency, we employed fReduce [49] and BioProspector [50]. As these software packages are intended to identify short sequence motifs associated with transcription factor binding sites, we substituted recombination frequency as faux expression data and inter-marker regions as promoter sequences. These analyses yielded no significant predictions, however one caveat is that rare motifs or highly degenerate motifs would be unlikely to be detected in this analysis due to the small size of the genome.

### An Engineered Hotspot

**Figure 5:**
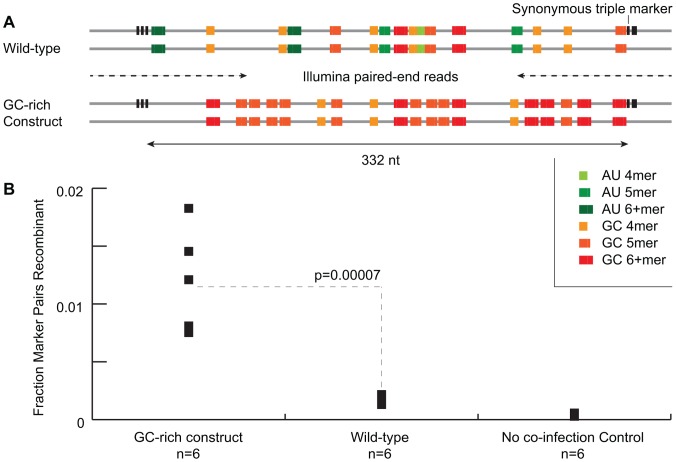
Creating a recombination hotspot. A. Wild-type poliovirus, with AU and GC tracts highlighted, was tagged with triple synonymous markers separated by 332 nt. Another construct was made by synonymously mutating bases to create or extend GC tracts and destroy AU tracts. An identical triple marker pair was installed on a derivative strain, both strains are shown with and without the triplet marker to stress that both pairs were assayed separately. B. Marked and unmarked viruses were co-infected and marker discordance calculated. The GC-rich construct, the wild-type strains and wild-type strains without co-infection as a control were assayed (n = 6 each).

The aggregated analyses revealed both secondary structure and AU/GC content as being significantly associated with bias in poliovirus recombination. To further validate and understand the relationship between GC- and AU-rich regions and recombination, we redesigned and synthesized a portion of the poliovirus capsid region with 40 synonymous mutations over a 332-nt region with the intention of creating or extending GC tetramers or disrupting AU tracts whenever possible. For this region, the number and length of GC tracts was increased (Table 2, Figure 5A) while AU tracts 4 nt or longer were eliminated. The GC content of the region was increased by 12% which resulted in a 26% increase in the overall predicted folding energy (−108.3 kcal/mol vs. −136.7 kcal/mol) (Unafold, M&M). This GC-rich construct was cloned into a wild-type poliovirus infectious clone. Synonymous mutations flanking the GC-rich region were added to both the test region construct and the wild type construct.

**Table 2:** Features of the GC-rich construct.

Feature	Wild-type	Construct
GC content	53.6%	65.7%
GC 4+ mers	11	19
GC 5+ mers	5	16
GC 6+ mers	2	7
GC 7+ mers	1	6
AU 4+ mers	5	0
AU 5+ mers	4	0
AU 6+ mers	2	0
AU 7+ mers	2	0
mfold energy (kcal/mol)	−108.3	−136.7
CpG elements	30	64
UpA elements	38	12

Coinfection experiments were performed and Illumina-compatible libraries generated for each virus pair as for the mapping experiment. This assay consisted of a single amplicon, requiring only a one-step RT-PCR in emulsion. Six coinfections each were performed with marked and unmarked wild-type virus, with the GC-rich construct and with no-coinfection controls of wild-type virus. The GC-rich construct was found to increase the rate of recombination by 7.4x over the 332-nt region (Figure 5B). This result supports our finding that the presence of GC-rich regions positively influences the rate of poliovirus recombination at those regions.

## Discussion

By combining synthetic poliovirus genome constructs with the large read depth conferred by Illumina sequencing, we describe a recombination map covering 82% of the poliovirus 1 coding region with over 50 thousand recombinant molecules observed. A whole genome recombination rate of 0.10 to 0.12 crossovers per genome per infectious cycle was observed for biological replicates. This rate is within the previously published estimates of 1–20% for near identical strains in cell culture [22], [23], [26]. It is important to note that our recombination estimate differs in form from most previous experiments by examining the RNA of all virions produced rather than examining viable isolates. We used a large number of input viral RNA genomes compared to observed genomes equivalents (50 million vs. 723 thousand) in order to minimize multiple observations of the same viral genome amplified by PCR with an estimated 87.5% recombinants observed being from unique input starting molecules. It is important to note that our estimates of recombination frequency and location are thus presented with the caveat that we have observed the frequency of recombinants at the conclusion of infection and not necessarily the recombination events themselves, as it is not possible to identify recombinant molecules that are phylogenetically unique. For example, a recombination event could be followed by replication and subsequent positive strand amplification, resulting in a bias that would not be distinguishable in this data.

This mapping technique is amenable to any virus for which there is an infectious clone and suitable cell line for transfection and coinfection, and could subsequently be applied to animal infections. Notably, this strategy is also possible in poorly studied viruses as no pair of selectable mutations need be identified and characterized prior to construct design. We note that this mapping strategy is intended for homologous recombination and is unsuited for the mapping of non-homologous recombination due to the reliance on specific PCR and amplicon size selection, which in turn selects against large deletions or duplications. While requiring a different methodology and controls, such maps should be feasible and comparison of those to homologous recombination maps could prove informative.

Poliovirus was used here as a well-understood model, but was also advantageous due to robust growth in cell culture. While our synthetic virus had an identical protein coding sequence to the wild type, there are presumably undiscovered RNA secondary structure elements in the poliovirus genome that were disrupted by the markers. Three mutations in the C1 strain arose, however none of these coincided with markers and thus cannot be considered direct revertants. Whether these mutations represent compensatory changes to currently unknown secondary structure elements or rose to prominence in the population for other reasons is unknown. It is important to note that there could be unanticipated selection forces operating within this mapping system that could result in bias for or against recombinant viruses. While it is impossible to eliminate the possibility, we have attempted to minimize the likelihood of such selection by avoiding the use of selectable markers and by collecting virus progeny after only one infectious cycle, conducted at high multiplicity of infection.

The sample preparation requirements of ultra-high throughput sequencing are prone to artifactual recombination by template switching during library production. Previous studies using RT-PCR to characterize recombination frequency may have avoided this issue by using extremely low starting concentrations of template. Library preparation techniques require quantities of template orders of magnitude greater than that required for RT-PCR, necessitating the development of the emulsion-based library generation protocol described here. We note that our emulsion generation method (bead milling) produces variable vesicle sizes that require generous template dilutions, and it is likely that this could be improved by utilizing microfluidic droplet makers [51]. Alternatively, Ozsolak et al [52] have sequenced RNA molecules directly without reverse transcription, which could provide a more direct means of assaying recombination with a similar viral construct design.

Phylogenetic studies rarely observe enterovirus recombinants with crossovers in the capsid region. This observation could be the result of protein incompatibility affecting viability, low nucleotide homology preventing recombination from occurring at all, or some sequence-based factor dampening recombination. Our results do not support a significant difference in recombination rate between the capsid and the non-structural region, even including the large hotspot at the RNAseL element.

The extremes of GC content, and in particular long tracts of only AU or GC nucleotides, are also associated with bias in recombination frequency. In the simplest interpretation, incomplete RNAs terminating in GC-rich sequences could be expected to anneal to a new template genome more robustly than AU-rich sequences as a straightforward matter of thermodynamics and in line with the established copy-choice mechanism (treated in King 1988 [29]). This interpretation suggests that in poliovirus, thermodynamic factors influence annealing of the nascent strand to the recipient genome to a greater extent than the initial dissociation of the donor genome. In the converse scenario, GC-rich regions would instead be less prone to fraying or dissociation from the original template and be associated with reduced recombination. The inverse symmetry of GC and AU effects further favors a simple thermodynamic model. An alternate and not exclusive model would consider RNA secondary structure to be the mechanism for recombination modulation, with GC and AU content influencing recombination indirectly by altering secondary structure stability.

Our results support earlier associations of the RNAseL element with recombination and further suggest that local secondary structure, as predicted *in silico*, also globally influences recombination rate. We also note that a recently described RNA secondary structure (Burril et al, personal communication) also corresponds to a recombination hotspot in the 3D region. While these two biologically functional secondary structures correspond to regions of high recombination, our *in silico* prediction simply examines the potential for local secondary structure, and not biological function. These conclusions suggest that it is plausible that a global redesign of the poliovirus genome could be implemented with the intent of reducing recombination potential by disrupting secondary structure elements and modulating nucleotide use.

**Figure 6:**
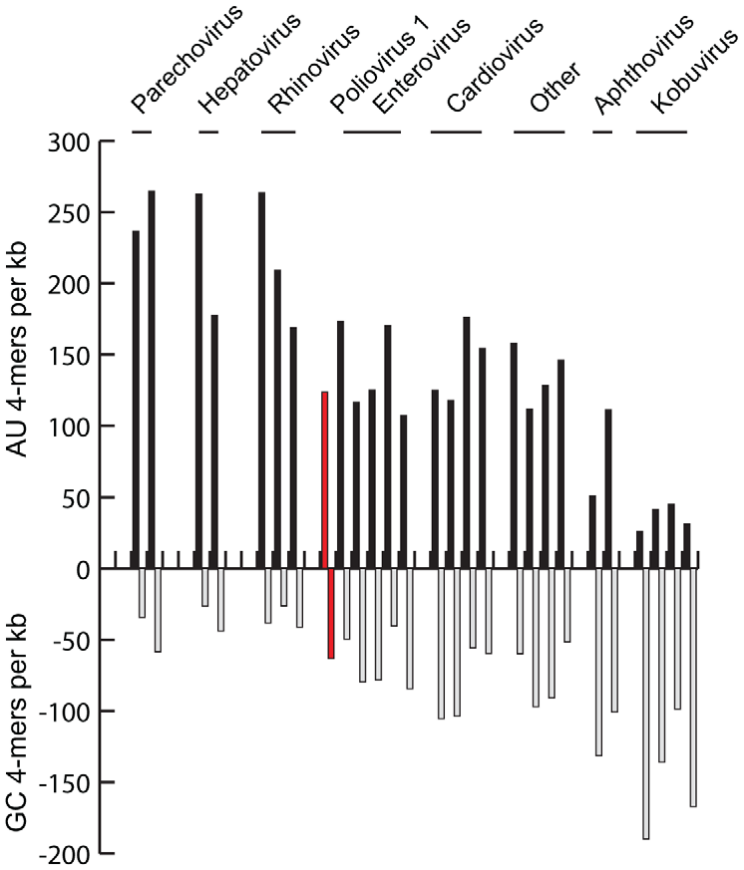
AU- and GC-tract frequency in Picornavirus species. Type strains of picornavirus species were analyzed for the presence of AU or GC 4-mers, with the absolute number of AU- or GC-tracts displayed on the positive and negative y-axes.

The frequency of AU and GC tracts is associated with the genomic GC content in Picornavirus species. Poliovirus represents a moderate case with a GC content of 46%. Other Enterovirus species, the genus Cardiovirus and most newly described or proposed genera have a similar GC content and AU/GC tract frequency (Figure 6). The genera Parechovirus, Hepatovirus and the Rhinovirus species all possess higher than average AU content, while the genera Apthovirus and Kobuvirus are GC rich relative to other picornaviruses. Based on the AU and GC tract associations described, we would predict that intra-typic homologous recombination rates within the GC-rich clades would be greater than poliovirus (eg. Aichivirus, FMDV), and that the AT-rich clades (parechoviruses, hepatoviruses, rhinoviruses) would have less intra-typic recombination potential than poliovirus. A major caveat of this prediction is that other factors, such as replication kinetics, the formation of replication rosettes, and differences in the viral polymerase could potentially confound such a simple relationship. Further, the tendency of each clade towards mixed heterotypic infections as a function of number of strains, shared cell tropism or frequency of infection are all confounding variables. No comparable recombination studies *in vitro* using nearly identical strains have been performed in these other picornaviruses, thus we cannot directly compare recombination frequency as opposed to other limits on homologous recombination.

The GC/AU and secondary structure motifs are straightforward to identify and can be engineered, with caveats. We modified a test region representing 4.5% of the genome to create or extend GC-rich tracts with synonymous mutations and eliminate AU tracts. The net effect of this modification was an increase in GC content (by 12%) and an increase in predicted folding energy (by 26%). This redesign underscores the difficulty of modifying coding sequence while leaving other, possibly vital, sequence factors in place. GC-content in virus sequences may be a form of adaptation to the host [53], [54] and it is possible that making GC-content changes across an entire genome will render a virus non-viable or adjust its growth parameters, such as cell tropism and permissive temperature. CpG and UpA elements in RNA are underrepresented in mammalian RNA viruses [55], [56] and have been associated with immune stimulation [57] and endonuclease susceptibility [58], [59]. Notably, Burns et al (2009) re-engineered Poliovirus 2 to increase GC content by 15% while maintaining CpG and UpA frequency without compromising viability in cell culture, however when only 9% of the genome was saturated with UpA and CpG elements the virus was rendered almost nonviable [36].

Lessons from poliovirus vaccines clearly teach the need for a better understanding of recombination potential and the factors that influence it. Ultimately, knowledge and manipulation of these factors may assist in the development and validation of recombination deficient attenuated vaccine strains.

## Methods

### Virus Design and Manipulations

Six different staggers are possible when synonymously recoding a sequence every 18-nt. A python script generated all possible staggers of the pAL-WT [60] plasmid containing a modified poliovirus 1 genome with the variant placing the fewest possible mutations on tryptophans or methionines, which cannot be synonymously mutated, selected for further redesign. A poliovirus codon table was used to mutate optimal codons to the second most optimal codon, and mutate all other codons to the optimal codon. When methionines or tryptophans were encountered, the marker was shifted one codon 5′ or 3′. Every ∼500 nt, sites of synonymous hyper-divergence were engineered with at least 5 mismatches within 9 consecutive nucleotides to act as specific primer sites for PCR- or qPCR-based low-resolution recombination assays. In addition, 22 single synonymous mutations were made to create unique restrictions sites in the infectious clone plasmid to facilitate future modification and RFLP assays. The design was submitted to Blue Heron (OriGene) for chemical synthesis. The construct infectious clone plasmid and pAL-WT were subsequently digested with BglII and ApaI (NEB), reciprocal fragments ligated and chemically transformed into Transformax cells (Epicentre) with a 30C overnight incubation step followed by subsequent bacterial culture at 37C (GenBank accessions JX286703-4).

Infectious clone plasmid DNA was linearized with MluI (NEB) prior to T7 *in vitro* transcription. 10 µg of RNA was electroporated in a 4 mm cuvette (300 V, 1000 µF, 24 Ohms) into 5×10^6^ HeLaS3 cells as a standard reaction; up to 50 µg of RNA was attempted for the construct cRNA (adapted from [60]). Cells were maintained in 50% DMEM/50% F12 media, 10% newborn calf serum and 2 mM glutamine; immediately after transfection cells were maintained in 10% Fetal Bovine Serum instead of NCS. Virus stocks were harvested after cytopathic effect (CPE) was observed by 3 rounds of freeze/thaw at −80C and 37C. Viruses were passaged at high Multiplicity of Infection (MOI) with a 1∶20 dilution of harvested media into fresh media and cells.

Plaque assays were performed on ∼10^6^ HeLaS3 cells in 6-well plates by washing cells with PBS, inoculation of 10-fold dilutions of virus in media, incubation for 60 minutes at 37C, an additional wash with PBS and overlay with 1% agarose and 50% DMEM/50% F12 with 1%NCS and 2 mM glutamine. One-step growth curves were performed in similar fashion with a 0.1 MOI virus inoculum and overlay in 10% NCS and media instead of an agarose formulation. Cultures were frozen at 2-hour intervals and harvested as above prior to plaque assay to determine viral load.

Coinfections were inoculated on 4×10^6^ cells with two virus stocks at an MOI of 10 each, washed after 1 hour and incubated for 24 hours in 10% NCS media prior to harvest and freeze-thaw. Viral RNA was extracted by Trizol (Invitrogen)/chloroform followed by isopropanol precipitation. Virus stocks for the competition assay were passaged at 0.1 MOI for an additional four passages. Competition assay RNA was amplified by non-strain specific primers, cloned by Topo-TA (Invitrogen) and colonies PCR amplified with strain specific primers to determine strain frequency. RT-qPCR was performed using primers PolioQF 5′-ACTCATTATCTATCTGTTTGCTGGATC and PolioQR 5′-TGATKGGCTCGGTGAACTTGG, with Superscript III reverse transcriptase (Invitrogen) and the Kapa 2x qPCR master mix (Kapa Biosystems) as per manufacturer's instructions on a Roche LC480 Lightcycler with an annealing temperature of 64C. RT-qPCR was used to determine viral RNA copy-number per mL of harvested culture supernatant and viral RNA concentration for input into the emulsion reverse transcription reaction, below.

### Emulsion Library Construction

Emulsion conditions were adapted from [40], emulsions were created by overlaying 600 µL of 2% EM90 (Degussa) and 0.05% Triton X-100 in light mineral oil (Sigma) with 200 uL of aqueous reaction mix on ice in 2 mL round-bottom tubes with 5 mm zinc-plated steel ball bearings. Solutions were shaken in a TissueLyzer II at 15 Hz for 10 sec and 17 Hz for 10 sec. Reactions were prepared in parallel to achieve a template occupancy ratio of 1∶1,000. 100 µL aliquots of emulsion were then transferred to 0.2 mL PCR tubes with a wide-bore pipette for thermocycling. For extraction, 100 µL of diethyl ether and 1 µL of 1% Cresol red (as an aqueous phase indicator dye) was added to each reaction and transferred to a 1.7 mL tube. PCR tubes were washed with an additional 100 µL of diethyl ether, which was also added to the recovery tube. Emulsions were broken by vortexing at maximum speed (3000 rpm) for 30 seconds and centrifugation at 13.2 k rpm for 1 minute followed by removal of the oil phase. This wash and breaking was repeated once with diethyl ether, once with ethyl acetate and then twice with diethyl ether. The aqueous phase was dried in a speed-vac centrifuge for 10 minutes and column purified (Zymo).

Reverse transcription and PCR reaction mixes were adapted to function under emulsion conditions: Bovine Serum Albumin (NEB) was added to a final concentration of 5% to serve as a bulking agent at the oil interface, detergent-containing reaction buffers were avoided and enzymes were added to 5% final reaction volume. All thermocyler incubation times were extended to at least 1 minute to facilitate heat transfer. Reverse transcriptions were performed with SuperScript II (Invitrogen) with manufacturer's buffers and PCR reactions performed with Phusion (NEB) with detergent-less High Fidelity buffer. Equivalent quantities of 25,000,000 genomes (10^5^ pg) of each virus were used in the reverse transcription reaction as determined by RT-qPCR and confirmed by BioAnalyzer (Agilent). The complete product of each step was then applied to each subsequent step. Reverse transcription was performed separately with three specific primers and each reaction was then amplified by PCR with the appropriate specific primer pair. Large PCR products were size-selected on a LabChip XT with the DNA 2 k beta chip and quantitated by BioAnalyzer. Products were then subjected to transposase-based library preparation by Nextera (Epicentre) followed by emulsion PCR with Phusion. The product of this reaction was size selected for 400–500 nt products using the LabChip DNA 750 chip, quantitated by qPCR (Kapa) and applied directly to sequencing on an Illumina HiSeq2000 with 100 nt paired end reads.

### Data Analysis

Deep sequencing data was filtered for quality: all sequences with more than 1 N were removed and sequences without a perfect match of at least 55 nt to either wild-type or construct strains were discarded. Reads were trimmed from 100 nt to 90 nt due to error rates of over 1% per base in the terminal region. Custom scripts were used to generate all possible recombinant and non-recombinant wild type and construct sequences spanning four markers (55 nt) and count perfect matches in the dataset. We identified an additional source of artifactual recombination that occurs during library preparation: both the RT and PCR steps utilize specific primer sites and at locations immediately 3′ of the primer sites (see PCR amplicons in Figure 2B) extremely high levels of apparent recombination were observed in both the no-infection control and experimental datasets. These false-recombinants presumably arose due to abortive initiation. We removed sites 40 nt 3′ of the primer sites from all subsequent analyses (3% of marker pairs). The ends of the PCR amplicons exhibited low read coverage and were also removed from this analysis (2% of marker pairs). Furthermore, the short region spanning the region of overlap between the two synthetic constructs was not covered by an amplicon in this analysis (6%). A total of 22 of 366 marker pairs were designed to either create or destroy a restriction site, providing target sites for RFLP assays of recombination. These marker pairs (6%) were also excluded from analysis.

Secondary structure predictions of the poliovirus genome were determined by Unafold [46] analysis of overlapping four-marker tiles (52 nt without the flanking markers). Other analysis platforms are discussed specifically in the text. The following models were considered for their presence between each marker pair: presence of a homopolymer of 4 nt or longer (4 models), presence of a dinucleotide tract of 4 nt or longer (6 models), or presence of a gene boundary (2 models). Non-binary models were considered by binning continuous scores into three similar size bins and attempting to associate the upper or lower bin vs the rest of the dataset (2 models each): GC content, LZW score, and unafold folding energy (over a 52 nt tile). In addition, two additional models were considered from the top output of the BioProspector and fReduce analysis packages for a total of twenty models; a multiple testing correction was applied to all association tests to compensate for this. Association tests were performed as Student's t-tests using the OpenEpi statistical calculator (www.openepi.com). Biological replicates were considered as discrete data points in this analysis, for a total of 580 marker pair data points.

### Calculations of Breakpoint Uniqueness

We coinfected 4×10^6^ cells with an MOI of 10 for each of two viruses (WT and construct). From the 1/10th volumes of supernatant extracted for RNA or plaque assayed, ∼3×10^8^ pfus or virions or ∼10^11^ encapisidated RNA genomes were recovered. We then applied 50,000,000 total genomes (25 million each) into the initial emulsion RT-step. The fraction of the input genomes that yielded full length 2.6 kb PCR products was approximately 5%, as determined by qPCR. Thus, the products of the emulsion PCR were derived from approximately 2.5 million starting molecules, comprising 725 million total marker pairs (290 marker pairs per genome equivalent). A total of 209.8 million marker pairs were mapped by sequencing. Assuming Poisson statistics, approximately 21% of the total marker pairs were sampled once, and approximately 3% were sampled more than once.

### Artificial Hotspot Experiment

A 400 nt DNA molecule was synthesized by IDT, added to a larger poliovirus PCR amplicon by fusion PCR and cloned into the prib(+)XpAlong [61] plasmid at restriction sites AatII and NheI. Triplet marker sites were added by modified primers amplifying construct or wild-type DNA, followed by similar fusion PCR and cloning steps. Viruses were generated and propagated from the infectious clones as above. The coinfection experiment was performed identically, however the library generation was executed in a single emulsion step using SuperScriptIII/Platinum Taq one-step RT-PCR mix (Invitrogen) and specific primers, otherwise as above. Amplicons were sequenced on a HiSeq2000 diluted to a ratio of <1∶10 with an unrelated insect RNA library to dampen decoupling effects; the poliovirus reads were prepared with unique DNA indices and were separated after sequencing. A lane that experienced severe over-clustering, which exacerbates the decoupling effect, was discarded from analysis.

## Data Submissions

Synthetic poliovirus constructs were submitted to GenBank (see materials and methods).

## Supporting Information

Figure S1 **Fitness characterization of construct strains.** A. One-step growth curves. Virus strains were applied to HeLa monolayers, washed and time-point samples frozen every two hours (x-axis) in triplicate (error bars). Samples were thawed and the titer measured by plaque assay (y-axis). B. Plaques formed by construct strains were not visually different from the wild type. C. Competition assay. Viruses were co-infected at equal titer, harvested and passaged into fresh cells four times. Viral RNA was extracted, amplified by strain-conserved primers, cloned and transformed into bacteria, and the relative quantity of each strain determined by strain specific colony PCR.
(EPS)

Figure S2 **Comparison of biological replicates.** HeLa monolayers were co-infected in parallel and proceeded through all steps of library preparation and sequencing separately. A. The recombination frequency at each marker pair is presented as a separate data point. Recombination was not observed at two data points; these are not included in the figure. B. Rank ordered list of marker pairs and corresponding recombination frequency.
(EPS)

Figure S3 **Comparison of experimental vs. no-coinfection control datasets.** A. Non-zero recombination frequencies are plotted comparing the experimental results with the control results to determine the similarity of artifactual recombination to biological recombination. B. Rank-ordered plot of experimental vs. control datasets, with the control x-axis expanded by 26-fold to display equivalent scale between the two datasets. A Pearson coefficient of r = 0.10 and a Spearman rank coefficient of ρ = 0.14 is observed between the datasets.
(EPS)

Table S1 **Infectivity characteristics of construct virus strains.** Construct viruses were assayed for viability compared to wild type by plaque-assay and qPCR for genomic RNA concentration. Burst size is calculated by total plaque forming units harvested divided by the number of cells infected.
(DOCX)
