# Supplementary material for: Identification and Manipulation of the Molecular Determinants Influencing Poliovirus Recombination
Source: PLoS Pathog. 2013 Feb 7;9(2):e1003164. doi: 10.1371/journal.ppat.1003164 (PMC3567174; doi:10.1371/journal.ppat.1003164)
Supplement: Table S1 — Infectivity characteristics of construct virus strains. Construct viruses were assayed for viability compared to wild type by plaque-assay and qPCR for genomic RNA concentration. Burst size is calculated by total plaque forming units harvested divided by the number of cells infected. (DOCX) [file ppat.1003164.s004.docx]

Table S1 Infectivity characteristics of construct virus strains

| Strain | pfu/mL | Burst Size | RNA genomes/mL | genome/pfu ratio |
| --- | --- | --- | --- | --- |
| Wild-type | 5.4-19.3 x 10^8^ | 405-1447 | 2.2-3.5 x 10^11^ | 116-645 |
| H1 | 3.4-12.0 x 10^8^ | 255-900 | 1.3-1.9 x 10^11^ | 104-573 |
| H2 | 2.9-6.1 x 10^8^ | 218-458 | 1.2-3.2 x 10^11^ | 192-532 |
| GC-rich construct | 3.2-6.6 x 10^8^ | 240-495 | 0.86-2.3 x 10^11^ | 131-712 |
